# A Dutch *MYH7* founder mutation, p.(Asn1918Lys), is associated with early onset cardiomyopathy and congenital heart defects

**DOI:** 10.1007/s12471-017-1037-5

**Published:** 2017-09-01

**Authors:** I. H. M. van der Linde, Y. L. Hiemstra, R. Bökenkamp, A. M. van Mil, M. H. Breuning, C. Ruivenkamp, S. W. ten Broeke, R. F. Veldkamp, J. I. van Waning, M. A. van Slegtenhorst, K. Y. van Spaendonck-Zwarts, R. H. Lekanne Deprez, J. C. Herkert, L. Boven, P. A. van der Zwaag, J. D. H. Jongbloed, M. Bootsma, D. Q. C. M. Barge-Schaapveld

**Affiliations:** 1 Department of Clinical Genetics, Leiden University Medical Centre, Leiden, The Netherlands; 2 Department of Cardiology, Leiden University Medical Centre, Leiden, The Netherlands; 3 Department of Paediatric Cardiology, Leiden University Medical Centre, Leiden, The Netherlands; 4 Department of Cardiology, Haaglanden Medical Centre, The Hague, The Netherlands; 5 Department of Clinical Genetics, Erasmus Medical Centre, Rotterdam, The Netherlands; 6 Department of Clinical Genetics, Academic Medical Centre, Amsterdam, The Netherlands; 7 University Medical Centre Groningen, Department of Genetics, University of Groningen, Groningen, The Netherlands

**Keywords:** Founder mutation, Cardiomyopathy, Congenital heart defect, Beta myosin heavy chain 7

## Abstract

**Background:**

Mutations in the myosin heavy chain 7 (*MYH7*) gene commonly cause cardiomyopathy but are less frequently associated with congenital heart defects.

**Methods:**

In this study, we describe a mutation in the *MYH7 *gene, c. 5754C > G; p. (Asn1918Lys), present in 15 probands and 65 family members.

**Results:**

Of the 80 carriers (age range 0–88 years), 46 (57.5%) had cardiomyopathy (mainly dilated cardiomyopathy (DCM)) and seven (8.8%) had a congenital heart defect. Childhood onset of cardiomyopathy was present in almost 10% of carriers. However, in only a slight majority (53.7%) was the left ventricular ejection fraction reduced and almost no arrhythmias or conduction disorders were noted. Moreover, only one carrier required heart transplantation and nine (11.3%) an implantable cardioverter defibrillator. In addition, the standardised mortality ratio for *MYH7* carriers was not significantly increased. Whole exome sequencing in several cases with paediatric onset of DCM and one with isolated congenital heart defects did not reveal additional known disease-causing variants. Haplotype analysis suggests that the *MYH7 *variant is a founder mutation, and is therefore the first Dutch founder mutation identified in the *MYH7* gene. The mutation appears to have originated in the western region of the province of South Holland between 500 and 900 years ago.

**Conclusion:**

Clinically, the p. (Asn1918Lys) mutation is associated with congenital heart defects and/or cardiomyopathy at young age but with a relatively benign course.

**Electronic supplementary material:**

The online version of this article (10.1007/s12471-017-1037-5) contains supplementary material, which is available to authorized users.

## Introduction

The myosin heavy chain 7 (*MYH7) *gene (OMIM, #160760) encodes a sarcomere protein. Mutations in *MYH7 *are a well-known cause of several types of cardiomyopathy, including hypertrophic cardiomyopathy (HCM) [1], dilated cardiomyopathy (DCM) [2], possibly occurring as peripartum cardiomyopathy [3], and left ventricular noncompaction, also referred to as noncompaction cardiomyopathy (NCCM) [4], in addition to skeletal myopathies with or without cardiac involvement [5]. Furthermore, several mutations in *MYH7* have been associated with congenital heart defects (CHDs), including Ebstein’s anomaly [6], bicuspid aortic valve (BAV) [7], and ventricular septal defect (VSD) [8], but thus far always in combination with cardiomyopathy (typically NCCM).

Several known pathogenic mutations in the *MYH7* gene are founder mutations, mutations that show a high frequency in a specific culturally or geographically isolated population. *MYH7* founder mutations have been described in South Africa [9], Finland [10], and Spain [11]. To the best of our knowledge, no Dutch *MYH7 *founder mutation has been reported to date.

We now describe a founder mutation in *MYH7*, c. 5754C > G; p. (Asn1918Lys), which is associated with several forms of cardiomyopathy with early or even prenatal onset in some patients, and with CHDs without cardiomyopathy even at advanced age.

## Methods

### Clinical evaluation

Clinical data were retrospectively collected on 15 probands (the first diagnosed case in a family) (A–O; family H was published previously [6]) and 65 of their family members, all of whom carried the *MYH7* p. (Asn1918Lys) mutation. All carriers were counselled at one of three academic centres (Leiden University Medical Centre, Erasmus Medical Centre Rotterdam or Academic Medical Centre Amsterdam).

Clinical information consisted of a medical and family history, physical examination, 12-lead electrocardiogram (ECG), echocardiography, exercise test and 24-hour (or 48-hour) Holter monitoring. If available, cardiac magnetic resonance imaging was also assessed. The cardiac phenotype was defined as either meeting criteria for a cardiomyopathy (defined as myocardial disease characterised by structurally and functionally abnormal myocardium and absence of other diseases, including congenital heart disease, sufficient to cause the observed myocardial abnormality) [12], and/or presence of a (corrected) CHD. Written informed consent was obtained from all participants as approved by local medical ethics committees.

### Genetic evaluation

Genomic DNA was extracted from peripheral blood samples using standard procedures. Probands were analysed between 2005 and 2016, initially by Sanger sequencing and then by next-generation sequencing using targeted gene panels [13]. Genetic variants were evaluated for their potential pathogenicity using *in silico* prediction tools, as available via the Alamut software (Interactive Biosoftware, Rouen, France).

Haplotype analysis was performed using 16 microsatellite markers around *MYH7*. DNA from 14 probands was analysed, and DNA samples of 6 family members carrying the mutation were used to verify the phase and reconstruct the haplotype. The age of the haplotype was based on the estimated number of generations since the mutation occurred [14], assuming 20–25 years per generation.

A Cytoscan HD SNP array (Affymetrix, Santa Clara, CA, USA) was performed on DNA from a carrier with a CHD (BAV) but without cardiomyopathy. DNA from this individual and three other carriers with childhood onset cardiomyopathy, and their parents, was used for whole exome sequencing (mean coverage ~100x). In brief, the exome was enriched with the Agilent Sureselect XT Human All Exon V5 kit followed by Illumina Hiseq2500 sequencing. The exome sequence was analysed using a stringent post-sequencing annotation pipeline (BWA-GATK-VEP) [15, 16]. Variant filtering and interpretation using a gene panel for CHDs of 81 genes (list available upon request: ldga@lumc.nl; including the *NOTCH1 *gene, known to be associated with BAV) was performed on the exome data of the first carrier using a custom-made version of the Leiden Open Variation Database (LOVDplus). For three childhood onset carriers, data files were uploaded to Cartagenia NGS bench (Cartagenia, Leuven, Belgium) and filtered using an automated filter tree. First, variants present in ~2,700 genes included in the Agilent SureSelect Inherited Disease (SSID) panel were selected. Next, variants in genes listed under the human phenotype ontology (HPO) term cardiomyopathy (HP:0001638; http://www.human-phenotype-ontology.org/) were selected, supplemented by genes absent from this HPO but otherwise associated with cardiomyopathy [17]. Interpretation was based on in-house lists available at the University Medical Centre Groningen, frequencies in population databases (ExAC, GoNL), in silico prediction programs (Alamut) and literature. Variants were reported as described (http://varnomen.hgvs.org/).

### Pedigree evaluation

Family pedigrees were constructed to determine presence or absence of the phenotype in mutation carriers and non-carriers (co-segregation). State archives, civil registers and online web trees from the west of the Netherlands were used to identify common ancestors. Postal codes of all carriers were plotted to determine the geographical distribution of the *MYH7* mutation.

### Statistical analysis

Descriptive statistics and the Mann-Whitney U test for median age of onset of cardiomyopathy (with interquartile range, IQR) per gender and per type of cardiomyopathy were performed using SPSS software (version 23.0, IBM Corp., Armonk, NY, USA). The standardised mortality ratio for *MYH7* carriers was estimated by utilising the mortality rate in the Dutch population in 2005 (https://www.cbs.nl/nl-nl/cijfers#theme=bevolking). Point estimate and corresponding 95% confidence intervals were calculated using Stata (StataCorp. 2015. Stata Statistical Software: Release 14. College Station, TX: StataCorp LP). Values of *p* < 0.05 were considered significant.

## Results

### Demographic data and risk factors

Of the 80 *MYH7* mutation carriers, 35 (44%) were male and the median age at testing was 47 years (IQR: 29.25–61). There were no significant differences in cardiovascular risk factors (hypertension, hypercholesterolaemia, diabetes and smoking) between carriers with a phenotype (either cardiomyopathy and/or CHDs, *n* = 49) and those without (*n* = 31). Two (6.5%) of the 31 carriers without a phenotype had symptoms of angina at the time of testing, one of whom was treated with percutaneous coronary intervention.

### Clinical diagnosis

#### Cardiomyopathy.

**Fig. 1:**
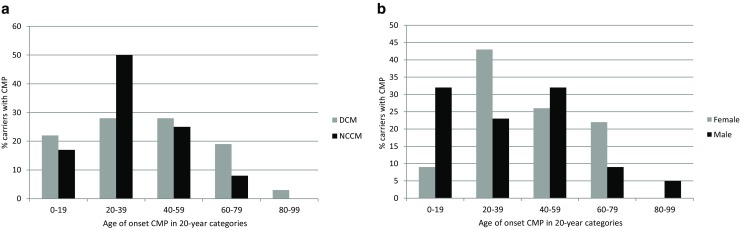
Histograms showing the percentage of carriers with cardiomyopathy by age of onset of cardiomyopathy (in 20-year categories): **a** per type of cardiomyopathy: DCM (*light upward diagonal lines*), NCCM (*black*). The only carrier with HCM is excluded; and (**b**) per gender: female (*light upward diagonal lines*), male (*black*)

Of the 15 probands, 9 met criteria for DCM, 5 for NCCM and 1 for HCM. Of the 65 family members, 31 were diagnosed with a cardiomyopathy, including 23 with DCM and 8 with NCCM, giving an overall penetrance for cardiomyopathy among the mutation carriers of 47.7%. Although eight family members were diagnosed with cardiomyopathy before the age of 12, overall median age of onset was 39 years (IQR: 25–56.5). There were no differences in age of onset per type of cardiomyopathy (40 years with IQR: 24.5–58 for DCM versus 30.5 years with IQR: 23.3–46.8 for NCCM, *p* = 0.38; the carrier with HCM was excluded, see Fig. 1a) nor per gender (32.5 years with IQR:7.25–54.25 for men versus 39 years with IQR:27–59 for women, *p* = 0.27; see Fig. 1b). In the female age group 20–39 years, 4 out of 10 had signs of peripartum cardiomyopathy.

#### Congenital heart defects.

Seven of 80 (8.8%) *MYH7* mutation carriers showed CHDs, including BAV (*n* = 4), BAV with coarctation of the aorta (*n* = 1), VSD (*n* = 1), and Ebstein’s anomaly (*n* = 1). In addition, two family members not tested for the mutation but with a 50% risk of being a carrier also had BAV (see individuals with (+), family D in Fig. 3). CHDs were confined to four of the 15 families (families A, C, D, and H in Fig. 3). The status of the aortic valve was not mentioned in the echocardiographic report of 12 *MYH7 *mutation carriers. Three carriers with BAV showed no cardiomyopathy, even though the youngest was 57 years of age. A cytoscan in one of these patients (see individual with (•), family D in Fig. 3) revealed no copy number changes and exome sequencing data showed no other mutation that might explain the CHD.

### Clinical characteristics

Over half of all probands (57.1%) showed a moderate to severely reduced left ventricular ejection fraction (LVEF, see Tab. 1), whereas the majority of family members with cardiomyopathy (63%) had mild to moderately reduced LVEF. Almost no arrhythmias or conduction disorders have been noted since the time of original testing. Six probands and three family members received an implantable cardioverter defibrillator (ICD). Of the six cases with available ICD records, only one had documented appropriate shock. One male family member underwent a heart transplant at age 50 and one female family member died at the age of 89 from heart failure, which was most likely due to ischaemia. The standardised mortality ratio was 1.19 (CI 95%: 0.74–1.80).

**Table 1:** Clinical characteristics of the *MYH7* p. (Asn1918Lys) mutation carriers

	Probands	Family members
Clinical diagnosis	(15)	(65)
– CMP	15 (100%)	31 (47.7%)
– CHD	1 (6.7%)	6 (9.2%)
Clinical measures	(14)	(27)
– LVEF		
∙ <60%	5 (35.7%)	17 (63.0%)
∙ <40%	8 (57.1%)	5 (18.5%)
– Arrhythmia/conduction		
∙ (P)AF	0 (0%)	2 (7.4%)
∙ Total AV block	0 (0%)	1 (3.7%)
∙ VT/VF	1 (7.1%)	0 (0%)
Outcome measures	(15)	(65)
– ICD implantation	6 (40%)	3 (9.6%)^a^
– Heart transplantation	0 (0%)	1 (3.2%)
– Death	0 (0%)	1 (3.2%)

*n* number of carriers with available clinical data, *CMP* cardiomyopathy, *CHD* congenital heart disease, *LVEF* left ventricular ejection fraction, (*P)AF* (paroxysmal) atrial fibrillation, *AV* atrioventricular, *VT/VF* ventricular tachycardia/ventricular fibrillation, *ICD* implantable cardioverter defibrillator

^a^Carrier with DDD-R pacemaker excluded

### Genetic diagnosis

The c. 5754C > G; p. (Asn1918Lys) variant in exon 39 of MYH7 (reference sequence NM_000257.3) was identified by Sanger or next-generation sequencing, and subsequently confirmed in the proband and family members by Sanger sequencing. The Asn1918 residue shows strong conservation between species, and the variant is located at the end of an important functional protein domain (coiled coil rod region). In addition, the variant was not identified in 170 ethnically-matched controls. However, as asparagine and lysine show no major differences in physicochemical properties (Grantham score: 94 [0–215]) the variant was initially classified as ‘likely pathogenic’, and was only reclassified as pathogenic following a report by Postma et al. in 2011 [5]. The variant is currently absent from >60,000 control exomes (ExAC database) (http://exac.broadinstitute.org).

### Geographical and historical origin of the mutation

#### Geographical distribution.

**Fig. 2:**
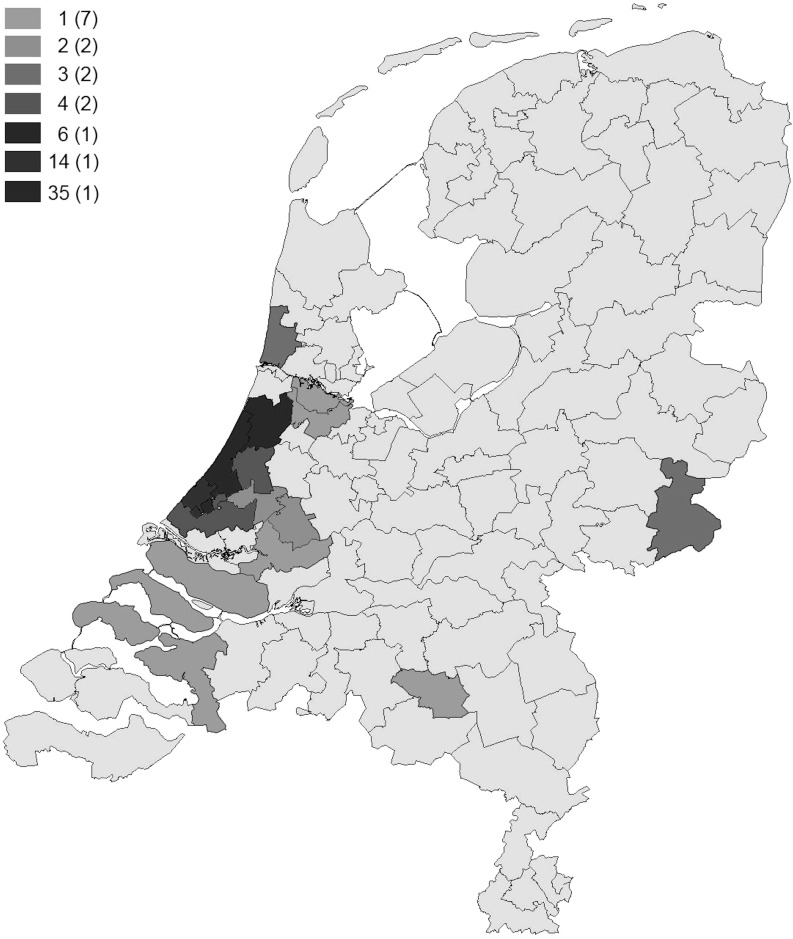
Postal code map. Number of carriers per postal code. Between brackets the number of postal code areas containing that specific number of carriers

Analysis of carrier postal codes (Fig. 2) showed that the mutation occurs mainly in the western part of the province of South-Holland. Despite this endemic location, no bi-allelic mutation carrier was encountered.

#### Genealogy.

**Fig. 3:**
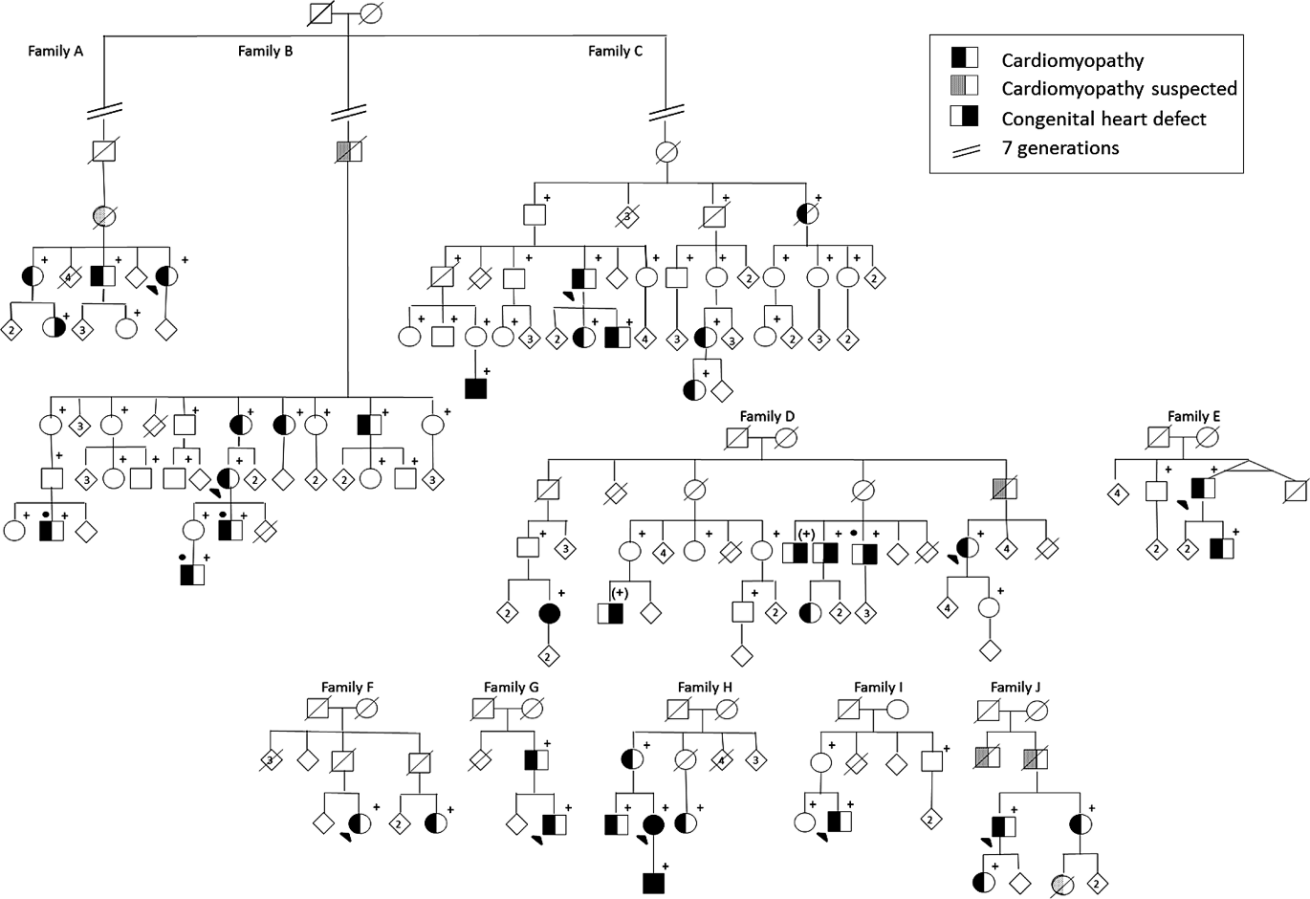
Pedigrees A through J (families of probands K–O are not shown as no family members are tested). Partly or fully filled symbols represent affected individuals (see box for explanation); (*open symbols* unaffected individuals, *diagonal line* deceased, *arrow* proband, *+* *MYH7* mutation carrier, *+* 50% risk for *MYH7* mutation, • tested with exome sequencing)

Genealogical research identified three pedigrees going back 11 generations (Fig. 3, families A, B and C) that were linked by a common ancestor around the mid-17th century.

#### Haplotype analysis.

A shared haplotype of at least two markers was found covering an 800 kb region surrounding *MYH7* (see Table 1 of the online Electronic Supplementary Material (ESM)). Based on haplotype data, we estimated that the *MYH7* mutation arose 25–36 generations ago and is 500–900 years old (assuming 20–25 years per generation).

### Genetic variability

#### Probands.

DNA variants in other genes (all classified as variants of unknown significance) were found in five probands tested in accordance with current practice in the Netherlands (core set of at least 45 genes). None of these DNA variants was shared (Online ESM Table 2, upper part).

#### Family members.

Exome sequencing in three family members (see individuals with (•), family B in Fig. 3) with an early age of onset (respectively prenatally, at the age of 1 and 7 years) found DNA variants of unknown significance in two cases. No variant was shared (Online ESM Table 2, lower part).

## Discussion

In this report, we identified 80 carriers of an *MYH7* founder mutation. Phenotypically, the *MYH7* p. (Asn1918Lys) mutation appears to be more closely associated with DCM, possibly as peripartum cardiomyopathy, or NCCM than HCM, as seen with other mutations in the tail region of the *MYH7 *gene [18]. *MYH7* mutations have occasionally been described in children with HCM [19] and with DCM [20], and even prenatally for DCM [21] or NCCM [22, 23]. However, an almost 10% prenatal or early childhood onset (<12 years) cardiomyopathy was unexpected. Interestingly, and despite the young age of onset of cardiomyopathy, this *MYH7* mutation appears to result in only mild to moderate clinical symptoms.

From a developmental point of view, the currently reported CHD (Ebstein’s anomaly, VSD, coarctation of the aorta and BAV) are all related to epicardial deficiencies [24] and, especially coarctation of the aorta, but also VSD and Ebstein’s anomaly, are known to be associated with BAV [25]. Although previous reports have described these CHDs in patients with specific *MYH7* mutations [4, 6–8, 26, 27] residing in the head and rod domain of the gene, p. (Asn1918Lys) is the only CHD-associated mutation that affects the tail region of the gene. Moreover, all previously described patients with an *MYH7* mutation and a CHD also had cardiomyopathy. The p. (Asn1918Lys) mutation is currently associated with CHDs in the absence of cardiomyopathy, although we cannot exclude that cardiomyopathy may develop later in life (>57 years).

The overall prevalence of CHDs in our cohort (8.8%) was higher than in the general population (1%) and was mainly explained by BAV (at least 6.3% in our patients versus 1.4% in the general population) [28]. Common epicardial deficiencies during development [24] might explain currently found CHDs in carriers of this *MYH7* mutation. However, as the CHDs were limited to four of the 15 families, other genetic or common environmental factors might be involved.

In families with inherited cardiomyopathies, DNA testing or regular cardiology examinations are advised from age 10 onwards. Based on current data, cardiac screening or genetic testing can be envisaged at an earlier age in children of a p. (Asn1918Lys) mutation carrier. In addition, at-risk couples should be advised of the need for prenatal echocardiography to detect CHDs. Furthermore, both our and other studies [29, 30] suggest a possible role for the *MYH7 *gene and other sarcomere genes (i. e. *MYH6*) in the development of CHDs. Inclusion of the *MYH7 *gene (and possibly other sarcomere genes) in current gene panels for CHDs should now be considered.

Our study design had several limitations. First, the retrospective design meant that follow-up data were not available for carriers under control elsewhere. Second, the follow-up period was limited to 11 years. Longer follow-up may improve cardiomyopathy penetrance estimates, especially in current CHD-only cases. Third, the numbers are too small for reliable estimation of the prevalence of CHD associated with this *MYH7* mutation. Finally, due to limitations in the genetic analysis of probands and family members, a role for other genes cannot be excluded.

In conclusion, this study describes the first Dutch founder mutation in the *MYH7* gene, a mutation associated with CHDs and cardiomyopathies, with frequent childhood onset but a relatively benign course.

## Caption Electronic Supplementary Material

Table 1. Shared haplotype surrounding the MYH7 gene in probands with p.(Asn1918Lys) mutation

Table 2. CMP phenotype and DNA variants found in set of genes tested for probands (upper part) and family members with early onset of DCM (lower part)
