# Supplementary material for: A Dutch MYH7 founder mutation, p.(Asn1918Lys), is associated with early onset cardiomyopathy and congenital heart defects
Source: Neth Heart J. 2017 Sep 1;25(12):675–81. doi: 10.1007/s12471-017-1037-5 (PMC5691818; doi:10.1007/s12471-017-1037-5)
Supplement: Supplementary file 1 — Table 1. Shared haplotype surrounding the MYH7 gene in probands with p.(Asn1918Lys) mutation [file 12471_2017_1037_MOESM1_ESM.docx]

**Table 1. Shared haplotype surrounding the *MYH7* gene in probands with p.(Asn1918Lys) mutation**

| **Marker** | **Pos** | **A** | | **B** | | **C** | | **D** | | **E** | | **F** | | **G** | | **H** | | **I** | | **K** | | **L** | | **M** | | **N** | | **O** | |
| --- | --- | --- | --- | --- | --- | --- | --- | --- | --- | --- | --- | --- | --- | --- | --- | --- | --- | --- | --- | --- | --- | --- | --- | --- | --- | --- | --- | --- | --- |
| D14S1003 | 21,6 | 161 | 161 | 161 | 163 | 161 | 163 | 161 | 163 | 161 | 161 | 159 | 163 | 161 | 161 | 161 | 163 | 161 | 159 | 161 | 161 | 161 | 159 | 161 | 163 | 161 | 163 | 161 | 161 |
| D14S50 | 21,8 | 171 | 177 | 171 | 171 | 171 | 173 | 171 | 177 | 175 | 177 | 173 | 177 | 173 | 177 | 171 | 171 | 171 | 179 | 171 | 175 | 171 | 177 | 175 | 175 | 171 | 177 | 171 | 177 |
| D14S283 | 22,2 | 121 | 133 | 121 | 121 | 121 | 129 | 121 | 129 | 127 | 149 | NA | NA | 133 | 141 | 121 | 127 | 121 | 133 | 121 | 133 | 121 | 133 | 133 | 129 | 121 | 133 | 121 | 133 |
| MYH7-1000K | 22,4 | 193 | 187 | 193 | 193 | 193 | 193 | 193 | 187 | 193 | 193 | 187 | 197 | 193 | 197 | 193 | 187 | 193 | 197 | 193 | 185 | 193 | 193 | 193 | 187 | 193 | 187 | 193 | 197 |
| MYH7-800K | 22,6 | 394 | 388 | 394 | 382 | 394 | 390 | 394 | 386 | 388 | 386 | 390 | 398 | 392 | 390 | 394 | 392 | 394 | 382 | 394 | 392 | 394 | 390 | 394 | 388 | 394 | 392 | 394 | 392 |
| MYH7-400K | 23,0 | 280 | 280 | 280 | 280 | 280 | 288 | 280 | 286 | 286 | 276 | 280 | 288 | 286 | 286 | 280 | 280 | 280 | 286 | 280 | 280 | 280 | 280 | 280 | 286 | 280 | 280 | 280 | 280 |
| MYH7 | 23,4 | + |  | + |  | + |  | + |  | + |  | + |  | + |  | + |  | + |  | + |  | + |  | + |  | + |  | + |  |
| D14S972 | 23,8 | 202 | 204 | 202 | 198 | 202 | 198 | 202 | 202 | 202 | 200 | 202 | 198 | 202 | 200 | 202 | 198 | 202 | 200 | 204 | 198 | 202 | 198 | 202 | 202 | 202 | 202 | 202 | 204 |
| D14S264 | 24,8 | 220 | 220 | 224 | 206 | 220 | 218 | 224 | 220 | 222 | 226 | 220 | 220 | 224 | 220 | 224 | 226 | 224 | 224 | 224 | 220 | 224 | 220 | 224 | 220 | 224 | 230 | 224 | 226 |
| D14S1032 | 25,7 | 256 | 260 | 258 | 258 | 260 | 264 | 268 | 272 | 258 | 266 | 270 | 272 | 268 | 268 | 268 | 276 | 268 | 268 | 258 | 258 | 256 | 260 | 268 | 268 | 268 | 274 | 268 | 276 |
| D14S275 | 26,2 | 197 | 197 | 193 | 191 | 191 | 191 | 193 | 197 | 193 | 197 | 191 | 193 | 193 | 195 | 193 | 191 | 193 | 193 | 193 | 195 | 193 | 193 | 193 | 193 | 193 | 191 | 195 | 197 |
| D14S80 | 27,2 | 148 | 152 | 148 | 144 | 152 | 146 | 144 | 146 | 138 | 138 | 144 | 146 | 148 | 146 | 148 | 142 | 148 | 140 | 148 | 146 | 148 | 138 | 148 | 148 | 148 | 146 | 148 | 148 |
| D14S262 | 29,0 | 197 | 199 | 197 | 201 | 199 | 199 | 195 | 201 | 195 | 203 | 195 | 199 | 197 | 195 | 195 | 195 | 197 | 195 | 199 | 201 | 197 | 195 | 195 | 201 | 197 | 195 | 195 | 199 |

Pos=position on chromosome 14 (Mb). NA=not available. The shared haplotype in 14 probands (J is not included) with the p.(Asn1918Lys) mutation is marked in grey. For patient K, the size of the first distal marker (D14S972) most likely changed due to slippage events during DNA replication. The same accounts for the size of marker D14S1032 in patients B, K and L.
