# Supplementary material for: A Dutch MYH7 founder mutation, p.(Asn1918Lys), is associated with early onset cardiomyopathy and congenital heart defects
Source: Neth Heart J. 2017 Sep 1;25(12):675–81. doi: 10.1007/s12471-017-1037-5 (PMC5691818; doi:10.1007/s12471-017-1037-5)
Supplement: Supplementary file 2 — Table 2. CMP phenotype and DNA variants found in set of genes tested for probands (upper part) and family members with early onset of DCM (lower part) [file 12471_2017_1037_MOESM2_ESM.docx]

**Table 2. CMP phenotype and DNA variants found in set of genes tested for probands (upper part) and family members with early onset of DCM (lower part).**

| **C** | **CMP**  **type** | **Genes tested**  **Reference sequence** | **MYH7 (NM_000257.3)** | **BAG3**  **(NM_004281.3)** | **DSC2**  **(NM_004949.4)** | **DSG2**  **(NM_001943.3)** | **DSP**  **(NM_004415.2)** | **PRDM16**  **(NM_022114.3)** | **VCL**  **(NM_014000.2)** | **FHL1**  **(NM_001159702.2)** | **SYNE1**  **(NM_182961.3)** |
| --- | --- | --- | --- | --- | --- | --- | --- | --- | --- | --- | --- |
| A | DCM | *MYH7, LMNA* | + |  |  |  |  |  |  |  |  |
| B | DCM | *MYH7, LMNA* | + |  |  |  |  |  |  |  |  |
| C | DCM | *MYH7, CSRP3, DES, LMNA, MYBPC3, TNNT2, TNNI3* | + |  |  |  |  |  |  |  |  |
| D | HCM | NGS (52 genes) | + | - | c.2194T>G,  p.(Leu732Val) | c.1174G>A,  p.(Val392Ile) | c.1778A>G,  p.(Asn593Ser) | - | - | - |  |
| E | DCM | NGS (60 genes) | + | - | - | - | - | c.2666C>T,  p.( Pro889Leu) | - | - |  |
| F | NCCM | NGS (48 genes) | + | - | - | - | - | - | - | - |  |
| G | NCCM | *MYH7* | + |  |  |  |  |  |  |  |  |
| H | NCCM | *MYH7* | + |  |  |  |  |  |  |  |  |
| I | DCM | NGS (23 genes) | + |  |  |  |  |  | - |  |  |
| J | DCM | NGS (48 genes) | + | - | - | - | - | - | - | - |  |
| K | DCM | NGS (55 genes) | + | - | - | - | - |  | c.688C>T,  p.(Arg230Cys) |  |  |
| L | DCM | NGS (60 genes) | + | - | - | - | - | - | - | - |  |
| M | DCM | *MYH7, MYBPC3* | + |  |  |  |  |  |  |  |  |
| N | NCCM | *MYH7, MYBPC3* | + |  |  |  |  |  |  |  |  |
| O | NCCM | NGS (47 genes) | + | c.1090G>A,  p.(Val364Met) | - | - | - | - | - | - |  |
| 1 | DCM | WES | + (m)  c.328G>A,  p.(Gly110Ser) (p) | - | - | - | - | - | - | c.968C>T.  p.(Pro323Leu) (m) | c.1827G>A,  p.(Glu6243Lys) (m) |
| 2 | DCM | WES | + (m) | - | - | - | - | - | - | - | - |
| 3 | DCM | WES | + (p) | - | - | - | - | - | - | - | - |

C = carrier (probands A to O; familymembers 1 to 3 with DCM at early age). + indicates the presence of the p.(Asn1918Lys) mutation in the *MYH7*gene. - indicates the absence of a variant in that specific gene, if tested. (m) maternally and (p) paternally inherited. WES = Whole Exome Sequencing (missense mutations in *TTN* gene are not reported). Minimal NGS (Next Generation Sequencing) set of genes: *Set1 =* *ACTC1, CSRP3, DES, GLA, LAMP2, LDB3, LMNA, MYBPC3, MYH7, MYL2, MYL3, PLN, PRKAG2, SCN5A, TAZ, TCAP, TNNC1, TNNI3, TNNT2, TPM1, VCL.* Extended set of genes: *Set2 =* *ACTN2, ANKRD1, BAG3, CALR3, CAV3, CRYAB, DSC2, DSG2, DSP, EMD, JPH2, JUP, LAMA4, MYH6, MYOZ2, MYPN, NEXN, PKP2, RBM20, TMEM43, TTN.*

NGS (23 genes) = *Set1* + *SGCD, STA.*

NGS (47 genes) = *Set1* + *Set2* + *CTNNA3* + *FHL1*+ *MIB1* + *PRDM16* + *TTR.*

NGS (48 genes) = NGS (47 genes) + *ABCC9*.

NGS (52 genes) = NGS (48 genes) + *ALPK3* + *NKX2.5* + *RYR2* + *TBX20*.

NGS (55 genes) = *Set1* + *Set2* + *ABCC9* + *DMD* + *DTNA* + *EYA4* + *GATAD1* + *MYOZ1* + *PSEN1* + *PSEN2* + *RYR2* + *SGCD* + *SOD2* + *TBX20* + *TXNRD2*.

NGS (60 genes) = NGS (48 genes) + *ANO5* + *DMD* + *DTNA* + *FKTN* + *HCN4* + *ILK* + *MYLK2* + *MYOZ1* + *RYR2* + *SGCD* + *TBX20* + *TXNRD2*.
